# “We Are All Trying to Find a Way to Help Ourselves”: A Look at Self-Help Strategies Among Psychotherapy Clients

**DOI:** 10.3389/fpsyg.2021.621085

**Published:** 2021-10-07

**Authors:** Sherilyn Chang, Rajeswari Sambasivam, Esmond Seow, Geoffrey Chern-Yee Tan, Sharon Huixian Lu, Hanita Assudani, Siow Ann Chong, Mythily Subramaniam, Janhavi Ajit Vaingankar

**Affiliations:** ^1^Research Division, Institute of Mental Health, Singapore, Singapore; ^2^Department of Mood and Anxiety, Institute of Mental Health, Singapore, Singapore; ^3^Department of Psychology, Institute of Mental Health, Singapore, Singapore

**Keywords:** self-help, psychotherapy, self-management, bibliotherapy, self-care

## Abstract

**Objective:** This study examined self-help strategies engaged by psychotherapy clients and explored their views on such self-help approach.

**Methods:** Secondary analysis of data from a qualitative research study was conducted. A total of 15 psychotherapy clients were recruited, and data were collected *via* semi-structured interviews. Thematic analysis of data was conducted using inductive approach to examine the content.

**Results:** Three main themes revolving around self-help strategies were identified: (1) types of self-help strategies, (2) reasons for engaging in self-help activities, and (3) effectiveness of self-help strategies.

**Conclusion:** The self-help approach to manage distress is common among psychotherapy clients. This study provided insights into understanding how and why clients use self-help strategies in their daily lives.

## Introduction

The use of self-help strategies to manage one’s own condition in the field of mental health has witnessed increase in popularity over the years (Norcross, 2000). In the context of treatments delivered through “health technology” such as computer programs, there is some differentiation between those conducted without any professional involvement (referred to as “pure” self-help) and those facilitated by a professional (“guided” self-help; Gellatly et al., 2007; Khan et al., 2007). Jorm and Griffiths (2006) used the term “informal self-help” to refer to “simple self-help that can be applied by the individuals affected without the need for professional guidance” and which can be used for early interventions in subclinical cases within the general population. For the purpose of discussion in this study, self-help is broadly defined and refers to any strategies or activities an individual does for the purpose of managing their distress or mental health in general. Such strategies could be self-initiated or acquired through learning materials disseminated by healthcare providers as part of supported self-management process. For instance, reading self-help books, attending support groups, practicing meditation, and/or accessing online health resources are some common examples. Individuals may choose self-help over professional help or supplement professional help because they cost less, are easier to access, and are less stigmatizing as compared to psychiatry-related services (Norcross, 2000; Marley, 2011).

A study by Jorm et al. (2000) found that “people with anxiety and depression symptoms rely primarily on simple self-help interventions, the effectiveness of which has been little researched.” The evidence surrounding the effectiveness of self-help interventions remains inconclusive with some studies finding self-help interventions effective in managing depressive (McKendree-Smith et al., 2003; Anderson et al., 2005; Gellatly et al., 2007) and anxiety symptoms (Lewis et al., 2012), while others suggesting that the evidence is limited (Bower et al., 2001; Coull and Morris, 2011). A recent review aimed at evaluating self-management interventions found support for such interventions in reducing symptoms among adults with severe mental illnesses and more importantly for personal recovery (van Weeghel et al., 2019), the increased sense of hope, empowerment, and self-efficacy from these interventions (Lean et al., 2019). Another study by Biringer et al. (2016) described the use of self-help strategies and environmental factors that facilitated recovery process among mental health service users.

In Singapore, a nationwide mental health literacy study found that the majority of the general population endorsed the usefulness of self-help practices (e.g., attending yoga or mediation classes, getting information on websites, reading up on people with similar experiences) for a person with mental disorder (Picco et al., 2016). Little is known, however, regarding self-help strategies adopted by psychotherapy clients in Singapore. This is important in understanding what mental health users do to cope with their distress, given the emphasis on empowering patients to play a greater role in their treatment and recovery (Mueser et al., 2002; Lucock et al., 2011). The aim of the present study was to examine the self-help strategies engaged by these individuals who were seeking psychotherapy treatment in a tertiary psychiatric hospital and to explore their views on such strategies.

## Materials and Methods

This is a secondary analysis of data from a qualitative study to examine concepts which were not central to the original research. The main aim of the primary study was to understand psychotherapeutic strategies and interventions to improve psychological wellbeing among psychotherapy clients.

### Setting and Recruitment

The primary study recruited participants attending outpatient psychotherapy at a tertiary psychiatric hospital in Singapore. Purposive sampling was employed, and recruitment posters were placed in the hospital clinic to inform patients about the ongoing study. Mental healthcare professionals (e.g., psychologists, psychiatrists, case managers) were also informed of the study who then referred interested participants who met the following study criteria: aged 21years and above, had attended at least two psychotherapy sessions in the past year, and able to provide consent. A total of 15 participants were enrolled for the study between January to October 2019, and one-to-one semi-structured interviews were conducted with them at the hospital or mutually agreed places such as cafes and workplaces.

### Study Procedures

Prior to the interview, participants first completed a short questionnaire to collect information on their sociodemographic background and clinical history. An interview guide which was designed in line with the primary aim to understand psychotherapeutic strategies to improve wellbeing of clients in the psychotherapy context was used for all interviews. Questions in the interview guide served as prompts and covered the following areas: recent experience with psychotherapy, ways to build positive mental health, improving positive mental health using psychotherapy, etc. Questions such as “what are some of the ways a person can improve their positive mental health?” and “have you heard or read about a therapy, for example, on the Internet?” led to participants volunteering information related to self-help strategies that is relevant for the present study. The interviews were conducted by two female researchers (JV and SC) with background in epidemiology and psychology and who have experience in qualitative research. All interviews were audio-recorded and transcribed verbatim with identifiers of the participants subsequently removed from the transcripts.

### Sample Characteristics

The sociodemographic profile of the participants (*n*=15) in this study is shown in Table 1. Participants in this sample mainly reported having depression and/or anxiety disorder (*n*=12), one with borderline personality disorder, and two were unaware of their clinical diagnosis. All participants were still attending psychotherapy at the time of interview, with the last session attended within 1month from the interview date. The types of therapy that participants reported receiving included cognitive behavioral therapy, mindfulness, movement desensitization and reprocessing, dialectical behavioral therapy, exposure and response prevention, schema therapy, acceptance and commitment therapy, and psychodynamic therapy; some participants were, however, unaware of the specific therapy they had received. The number of psychotherapy sessions attended in the past 1year by these participants ranged from 2 sessions to 48 sessions (median=7.5).

**Table 1:** Profile of participants (*n*=15).

		*N*
Gender	Males	6
	Females	9
Age group	21–39	10
	40–65	5
Ethnicity	Chinese	9
	Malay	4
	Indian	2
Marital status	Single	11
	Married	3
	Separated	1
Education	Secondary	2
	Vocational and diploma	6
	Tertiary and above	7
Employment	Employed	4
	Not employed	11

### Analysis of Data

Thematic analysis of the data in the original study and current study was conducted using inductive approach (Braun and Clarke, 2006). This approach was chosen as it is a flexible technique to be used across theoretical frameworks and that there are no pre-existing concepts used in this study, particularly given the interest of this study was to explore and gain cross-sectional insights into experiences of psychotherapy clients in general. It is also appropriate for use in this secondary analysis of data given the good fit of the purpose and aim of this analysis with the primary study, and it allows rich description of the data set.

The coding team consisted of four researchers of which three were females (JV, RS, and SC) and one was male (ES) and all with background in epidemiology and psychology. While JV and SC conducted the interviews, all coding members were also involved in transcription and checking accuracy of the transcripts. In the initial coding stage, four transcripts were randomly selected and read individually by the coding team to independently code for important and relevant content, and the codes were combined to generate a list of preliminary themes. This list was reviewed, and subsequently emergent themes were formed following discussions within the team. The remaining transcripts were coded, and new codes were added when necessary to capture novel content, and the emergent themes were subsequently modified after multiple deliberations. Data related to the theme on self-help were extracted for this study, and one member (SC) led the analysis and inductively combined the codes and emergent themes based on their interrelation and co-occurrence to produce higher-order themes, which were reviewed and discussed by the other three members. Disagreements were resolved through discussion within the team members to reach consensus.

## Results

Three main themes revolving around self-help strategies were identified: (1) types of self-help strategies, (2) reasons for engaging in self-help activities, and (3) effectiveness of self-help strategies.

### Types of Self-Help Strategies

All participants, except for two, reported using a range of self-help activities and strategies to manage their mental health problems. These were broadly classified into two categories: generic wellbeing promotion activities and problem-focused strategies. The former pertains to activities that are generally regarded as enhancing overall health and mental wellbeing, for instance participants reported engaging in physical activities and hobbies, talking to friends, expressing of self through art, and maintaining a routine (e.g., completing house chores). A number of participants also mentioned reading self-help books and watching online motivational videos.

Problem-focused strategies, by contrast, are in relation to participant’s existing mental health condition or psychological concerns and thus represent more targeted actions. Participants described searching for information and reading up on their condition and strategies taught by their therapist, and one participant talked about attending mental health workshops. Some of these strategies were initiated by the therapist and used by clients beyond the therapy session, for instance practicing psychotherapeutic techniques such as mindfulness strategies, breathing exercises, and positive visualization. Some participants described self-monitoring of behavior and mood with the aid of mobile applications, for instance using it to track alcohol consumption.

### Reasons for Engaging in Self-Help Activities

Participants described underlying psychological reasons and motivations for engaging in self-help activities. These included the desire to seek changes in attitudes and behavior, to acquire knowledge of symptoms, and the value of self-care (Table 2).

**Table 2:** Reasons for engaging in self-help behaviors.

Seek changes in attitudes and behaviors	*Attitude* “So for ‘Road Less Travelled’ (book) it help quite a bit. I remember… ‘if you are not part of the solution then you are part of the problem.’ It might sound quite harsh but the essence is so good you know, if you want to improve anything rather than you complain about it, you just be part of the change you see. So for me, I also apply that for myself.” S1/F/46yo “I can use those strategies, use those mindfulness techniques to help myself become a more positive person.” S2/M/22yo “That kind of video when I watch right, I feel wow these people have such a strong will to survive… this kind of videos help me to value life, help me to really think, wow how these people they are so strong you know I mean they are even in the face of death you know they want to live.” S13/F/55yo *Behavior* “I will use that app to like do breathing exercises and to just help me fall asleep.” S3/F/40yo “I think little things like this where I sit down and I trim my nails and I go for a run…I know that running produces endorphins and it helps the chemical systems and I think it’s, I think given that space and that distance away from certain stressful situations.” S5/M/32yo “…the self-help book says participate in class. Of course, if you do not have confidence, you dare not. But I try to do it. So I participate more in class…” S6/F/49yo
Acquire knowledge on symptoms	“Before this I wasn’t aware of the mental issues... So I went to few workshops about mental health so that helped me, like the way I’m feeling, passion all this… I had that knowledge then I can see my own depression in myself and other people…” S8/M/24yo “Then I got a lot of information from reading. Then it seems like I tick all the boxes I have all the symptoms you know of a depressive person.” S13/F/55yo
Value self-care	“I am my own caregiver. I have to take care of myself, monitor my illness, I monitor my mood.” S14/M/42yo “Helping yourself is much- because people think that, everybody else is not understanding, they are not helping me. But actually, you are the person that actually need to help yourself.” S15/F/35yo

Aside from psychological motivations for using self-help strategies, practical reasons for using certain self-help strategies were also mentioned by the participants. Customizability of activities and flexibility in choosing them according to the participants’ beliefs, needs, and interest were some of these reasons. Others included self-help activities being chosen as alternatives to psychotherapy owing to factors such as cost (“…I was aware of psychotherapy… but because it’s too expensive, then I went to books”; S1/F/46yo) or perceived ineffectiveness of therapy sessions (“…I have to go for the therapy, then it did not work out… so over the years I try find out my own triggers. I find the way to isolate. I find a way to contain”; S11/M/28yo).

### Effectiveness of Self-Help Strategies

Self-help strategies were effective and notably helpful for several participants in changing their behavior (e.g., improvement in sleep quality) and attitudes (e.g., developing positive outlook in life). Age and employment status, however, were factors described by participants which had affected their level of comprehension and participation for certain self-help strategies. One participant (S1/F/46yo) recalled reading a self-help book that “did not make any sense” as a youth, but it was “so helpful” when she came across it again as a young adult. Another participant noted that being unemployed has given him “the time and space to pursue these things (self-help activities)” (S5/M/32yo).

Some participants noted difficulties they had encountered in the process of engaging in self-help activities. One participant (S1/F/46yo) pointed out potential “blind spots” in self-help activities where the support obtained “may not necessarily go to the core” of their presenting issues. Another participant alluded to the need to be self-reliant or independent as a challenge when practicing self-help bibliotherapy – “I find that reading on my own… having the book telling you what to do… it’s different from when someone tells me to do it and someone practices it with me there on the spot” (S3/F/40yo). Even for strategies introduced by the therapist, taking on the self-help approach was nonetheless described by one participant as something that “takes a lot of effort… because the therapist only can tell me what to do, then I have to execute” (S8/M/24yo). Similarly, another participant mentioned that he lacked the “courage to use the [inter]net” for self-help, despite having practiced some of the strategies with the therapist (S2/M/22yo). There was concern about the lasting effect of self-help strategies (“But once the practice session stops, then it’s sort of like I keep worrying about things again”; S7/F/35yo).

## Discussion

This study provided a preliminary understanding of self-help strategies used by psychotherapy clients and of their views of such self-help approaches. Clients described their motivations for using self-help strategies as a way to achieve attitudinal and behavioral changes. This could be one of the ways to attain some form of control in their lives and to take charge in managing their distress (Mental Health Foundation, 2000). Parallels can also be drawn from another study which identified empowerment and managing and structuring the day as useful self-help approaches individuals engaged in for day-to-day problems (Lucock et al., 2007). These themes highlight the capacity of clients to be able to proactively participate in or plan activities that can influence their wellbeing and bring about positive changes. Additionally, the study by Lucock et al. (2007) also noted that empowerment could be derived from possession of information, which resonates with the finding in this study where the participants reported the need to gain knowledge of symptoms as a reason for adopting self-help behaviors.

These findings support the notion that engaging in self-help strategies promotes a sense of self-efficacy among individuals with mental distress (Jorm and Griffiths, 2006). This self-belief in being able to handle situations and exert control was also reported by clients who engaged in self-therapeutic activity after completion of therapy with a mental healthcare professional (Glasman et al., 2004). Clients described it as their “responsibility” to deal with relapses that occurred. In a way this also reflects the value and importance that clients place on self-care and hence the reason for taking on the self-help approach to manage their conditions, as echoed by clients in this study.

While it is unclear whether some of the self-help strategies were in fact mandatory “homework” assigned by therapist as a part of the course of treatment, which there are then implications on clients’ willingness to be proactive in taking steps for their recovery, it may still be beneficial for clients to explore the self-help approach, given the successful outcomes reported by several clients in this study. These strategies could potentially supplement ongoing therapeutic interventions that clients have with their mental healthcare providers. Furthermore, these self-help practices could be important coping strategies when it is difficult to have face-to-face sessions as in the lockdown imposed during this COVID-19 pandemic which have disrupted outpatient visits for individuals seeking mental health treatment (Yao et al., 2020).

However, as described by clients in this study and that by Glasman et al. (2004), the self-help approach can be “effortful” and requires “hard work,” even for strategies initiated by their therapists. This study also noted several other difficulties that clients encountered when engaging self-help practices. As the present study reports findings from a secondary analysis of data, a limitation of the study was that analysis was restricted to the data originally collected, and hence, many of the issues and difficulties with the self-help approach could not be further explored. A recommendation for future research would be to understand how these difficulties can be addressed and explore ways to best render support to clients for such self-help approach to work. Furthermore, it would be meaningful to profile clients to understand their self-help preferences and also to identify common aspects of self-help strategies that are associated with positive outcomes for the clients. Understanding these issues will provide valuable insights for mental health professions to empower clients to adopt the self-help strategies both concurrently, and also separate from, ongoing psychotherapy.

More importantly, future studies should look into evaluating the effectiveness of self-help practices among psychotherapy clients. While clients in this study described effectiveness and challenges they have encountered, it was at times an overview of self-help approach in general rather than in relation to a specific type of strategy. Future studies may look into exploring and understanding potential links between effectiveness and the type of self-help strategies, as well examining it with respect to the underlying motivations. This was also identified by service users as one of the priorities for research into self-help to understand what works and why (Lucock et al., 2007). Given that the use of non-evidence-based self-help was found to have negative impacts on users (Yeung and Lun, 2020), for example, among individuals with low self-esteem (Wood et al., 2009), the effectiveness of self-help strategies ought to be communicated to clients so that they understand the boundaries and limits of which these self-help activities work. While they may supplement and act as an adjunct to therapy, general advices from self-help sources could potentially be misinterpreted by clients and leading to wrong or blind application that sets them up for disappointment.

Nonetheless, this study represents pioneer work to understand self-help strategies engaged by psychotherapy clients in Singapore. It is evident from this study that psychotherapy clients proactively partake in various self-help activities with differing underlying psychological and practical motivations and to varying levels of effectiveness experienced. Findings provided insights for healthcare professionals on understanding strategies that psychotherapy clients adopt and ways in which they are empowered to manage their distress. After all, the self-help approach is common, as one client aptly pointed out: “we are all trying to find a way to help ourselves.”

## Data Availability Statement

Data is not available for online access, however, readers who wish to gain access to the data can write to the senior author JV at janhavi_vaingankar@imh.com.sg with their requests. Access can be granted subject to the institutional review board (IRB) and the research collaborative agreement guidelines. This is a requirement mandated for this research study by our IRB and funders.

## Ethics Statement

The studies involving human participants were reviewed and approved by the National Healthcare Group Domain Specific Review Board. The patients/participants provided their written informed consent to participate in this study.

## Author Contributions

JV, MS, and SAC were involved in the conception and design of the study with expertise inputs from GC-YT, SL, and HA. SC, RS, ES, and JV were involved in interpretation of the data, recruitment, and conducting of qualitative interviews, and undertook the analysis of the data. SC drafted the manuscript under the supervision of JV. All authors contributed to the article and approved the submitted version.

## Funding

The study was funded by the Singapore Ministry of Health’s National Medical Research Council under the Centre Grant Programme (NMRC/CG/M002/2017).

## Conflict of Interest

The authors declare that the research was conducted in the absence of any commercial or financial relationships that could be construed as a potential conflict of interest.

## Publisher’s Note

All claims expressed in this article are solely those of the authors and do not necessarily represent those of their affiliated organizations, or those of the publisher, the editors and the reviewers. Any product that may be evaluated in this article, or claim that may be made by its manufacturer, is not guaranteed or endorsed by the publisher.

## References

[ref1] Anderson L. Lewis G. Araya R. Elgie R. Harrison G. Proudfoot J.. (2005). Self-help books for depression: how can practitioners and patients make the right choice? Br. J. Gen. Pract. 55, 387–392. PMID: 15904559 PMC1463163

[ref2] Biringer E. Davidson L. Sundfør B. Lier H. Ø. Borg M. (2016). Coping with mental health issues: subjective experiences of self-help and helpful contextual factors at the start of mental health treatment. J. Ment. Health 25, 23–27. doi: 10.3109/09638237.2015.1078883, PMID: 26484831 PMC4776697

[ref3] Bower P. Richards D. Lovell K. (2001). The clinical and cost-effectiveness of self-help treatments for anxiety and depressive disorders in primary care: a systematic review. Br. J. Gen. Pract. 51, 838–845. PMID: 11677710 PMC1314132

[ref4] Braun V. Clarke V. (2006). Using thematic analysis in psychology. Qual. Res. Psychol. 3, 77–101. doi: 10.1191/1478088706qp063oa

[ref5] Coull G. Morris P. G. (2011). The clinical effectiveness of CBT-based guided self-help interventions for anxiety and depressive disorders: a systematic review. Psychol. Med. 41, 2239–2252. doi: 10.1017/S0033291711000900, PMID: 21672297

[ref6] Gellatly J. Bower P. Hennessy S. Richards D. Gilbody S. Lovell K. (2007). What makes self-help interventions effective in the management of depressive symptoms? Meta-analysis and meta-regression. Psychol. Med. 37, 1217–1228. doi: 10.1017/S0033291707000062, PMID: 17306044

[ref7] Glasman D. Finlay W. Brock D. (2004). Becoming a self-therapist: using cognitive-behavioural therapy for recurrent depression and/or dysthymia after completing therapy. Psychol. Psychother. 77, 335–351. doi: 10.1348/1476083041839385, PMID: 15355585

[ref8] Jorm A. F. Griffiths K. M. (2006). Population promotion of informal self-help strategies for early intervention against depression and anxiety. Psychol. Med. 36, 3–6. doi: 10.1017/S0033291705005659, PMID: 16356291

[ref9] Jorm A. F. Medway J. Christensen H. Korten A. E. Jacomb P. A. Rodgers B. (2000). Public beliefs about the helpfulness of interventions for depression: effects on actions taken when experiencing anxiety and depression symptoms. Aust. N. Z. J. Psychiatry 34, 619–626. doi: 10.1080/j.1440-1614.2000.00761.x, PMID: 10954393

[ref10] Khan N. Bower P. Rogers A. (2007). Guided self-help in primary care mental health: meta-synthesis of qualitative studies of patient experience. Br. J. Psychiatry 191, 206–211. doi: 10.1192/bjp.bp.106.032011, PMID: 17766759

[ref11] Lean M. Fornells-Ambrojo M. Milton A. Lloyd-Evans B. Harrison-Stewart B. Yesufu-Udechuku A.. (2019). Self-management interventions for people with severe mental illness: systematic review and meta-analysis. Br. J. Psychiatry 214, 260–268. doi: 10.1192/bjp.2019.54, PMID: 30898177 PMC6499726

[ref12] Lewis C. Pearce J. Bisson J. I. (2012). Efficacy, cost-effectiveness and acceptability of self-help interventions for anxiety disorders: systematic review. Br. J. Psychiatry 200, 15–21. doi: 10.1192/bjp.bp.110.084756, PMID: 22215865

[ref13] Lucock M. Barber R. Jones A. Lovell J. (2007). Service users’ views of self-help strategies and research in the UK. J. Ment. Health 16, 795–805. doi: 10.1080/09638230701526521

[ref14] Lucock M. Gillard S. Adams K. Simons L. White R. Edwards C. (2011). Self-care in mental health services: a narrative review. Health Soc. Care Community 19, 602–616. doi: 10.1111/j.1365-2524.2011.01014.x 21749527

[ref15] Marley E. (2011). Self-help strategies to reduce emotional distress: what do people do and why? A qualitative study. Couns. Psychother. Res. 11, 317–324. doi: 10.1080/14733145.2010.533780

[ref16] McKendree-Smith N. L. Floyd M. Scogin F. R. (2003). Self-administered treatments for depression: a review. J. Clin. Psychol. 59, 275–288. doi: 10.1002/jclp.10129, PMID: 12579545

[ref17] Mental Health Foundation (2000). Strategies for Living Summary. Available at: https://www.mentalhealth.org.uk/publications/strategies-living-summary (Accessed April 27, 2020).

[ref18] Mueser K. T. Corrigan P. W. Hilton D. W. Tanzman B. Schaub A. Gingerich S.. (2002). Illness management and recovery: a review of the research. Psychiatr. Serv. 53, 1272–1284. doi: 10.1176/appi.ps.53.10.1272, PMID: 12364675

[ref19] Norcross J. C. (2000). Here comes the self-help revolution in mental heath. Psychother. Theory Res. Pract. Train. 37, 370–377. doi: 10.1037/0033-3204.37.4.370

[ref20] Picco L. Abdin E. Chong S. A. Pang S. Vaingankar J. A. Sagayadevan V.. (2016). Beliefs about help seeking for mental disorders: findings from a mental health literacy study in Singapore. Psychiatr. Serv. 67, 1246–1253. doi: 10.1176/appi.ps.201500442, PMID: 27524364

[ref21] van Weeghel J. van Zelst C. Boertien D. Hasson-Ohayon I. (2019). Conceptualizations, assessments, and implications of personal recovery in mental illness: a scoping review of systematic reviews and meta-analyses. Psychiatr. Rehabil. J. 42, 169–181. doi: 10.1037/prj0000356, PMID: 30843721

[ref22] Wood J. V. Elaine Perunovic W. Q. Lee J. W. (2009). Positive self-statements: power for some, peril for others. Psychol. Sci. 20, 860–866. doi: 10.1111/j.1467-9280.2009.02370.x, PMID: 19493324

[ref23] Yao H. Chen J.-H. Xu Y.-F. (2020). Patients with mental health disorders in the COVID-19 epidemic. Lancet Psychiatry 7:e21. doi: 10.1016/S2215-0366(20)30090-0, PMID: 32199510 PMC7269717

[ref24] Yeung J. C. Lun V. M.-C. (2020). Uncritical use of non-evidence-based self-help materials induces victim-blaming on depressed individuals. J. Posit. Psychol. 16, 492–502. doi: 10.1080/17439760.2020.1752780

